# Phytochemical Screening, Physicochemical Properties, Acute Toxicity Testing and Screening of Hypoglycaemic Activity of Extracts of *Eremurus himalaicus* Baker in Normoglycaemic Wistar Strain Albino Rats

**DOI:** 10.1155/2014/867547

**Published:** 2014-04-29

**Authors:** Ahlam Mushtaq, Seema Akbar, Mohammad A. Zargar, Adil F. Wali, Akhtar H. Malik, Mohammad Y. Dar, Rabia Hamid, Bashir A. Ganai

**Affiliations:** ^1^Department of Biochemistry, University of Kashmir, Srinagar, Jammu and Kashmir 190006, India; ^2^RRIUM, University of Kashmir, Srinagar, Jammu and Kashmir 190006, India; ^3^Department of Pharmaceutical Sciences, University of Kashmir, Srinagar, Jammu and Kashmir 190006, India; ^4^Centre for Biodiversity and Taxonomy, Department of Botany, University of Kashmir, Srinagar, Jammu and Kashmir 190006, India

## Abstract

In the present study EtOAc, MeOH, and aqueous extracts of *Eremurus himalaicus* were evaluated for hypoglycaemic effect in normal rats using both oral glucose tolerance test and 14-day oral administration study. Phytochemical and physicochemical screening was also done. In oral glucose tolerance test the aqueous and MeOH extracts of *Eremurus himalaicus* at a dose level of 500 mg/kg body weight prior to glucose load resulted in a significant fall in blood glucose level within 150 min. of glucose administration. The aqueous extract at a dose level of 250 mg/kg body weight and 500 mg/kg body weight also showed good hypoglycaemic response (*P* < 0.001); this was followed by MeOH extract at a dose level of 500 mg/kg body weight (*P* < 0.05), while MeOH extract at dose level of 250 mg/kg body weight and ethyl acetate extract at dose level of 250 mg/kg body weight and 500 mg/kg body weight exhibited insignificant effect. Phytochemical screening of extracts revealed the presence of alkaloids, terpenoids, phenolics, tannins, saponins, cardiac glycosides, and flavonoids. The results indicate that aqueous extract possess significant hypoglycaemic activity in normoglycaemic rats which may be attributed to the above-mentioned chemical constituents.

## 1. Introduction

Diabetes, a global burden, is characterized by fast elevation of blood sugar level. The incidence of diabetes mellitus is rising all over the world, especially in Asia. Many oral hypoglycaemic agents, such as biguanides and sulfonylureas are available along with insulin for the treatment of diabetes mellitus, but they have significant side effects and sometimes they are found to be ineffective in chronic diabetic patients [1, 2]. Thus, there is an increasing demand of natural and synthetic products with high antidiabetic potential and lesser side effects. The research conducted over the last several decades has shown that plant and plant-based therapies have high potential to treat and control diabetes and its complications [3]. Diabetes has been treated orally with several medicinal plants or their extracts, based on folklore medicine. Therefore, search for safe and more effective agents has continued to be an important area of active research.

Looking back upon the last 2000 years of the history of medicine mankind has mainly used plants as the best source of medicine [4]. Over 248,000 species of higher plants have been identified and from these 12,000 plants are known to have medicinal properties [5]. The significance of research on natural medicinal plants is gaining momentum owing to its immense potential for improving the health care sector of the globe. The World Health Organization estimated that about 80% of Earth's inhabitants rely on traditional medicine for their primary health care needs that primarily involves the use of plant extracts or their active components. A concerted research is currently in vogue for understanding the mechanism of action of these traditional procedures. With the sharp rise in popularity of traditional medicine, the economic importance of these plants has increased enormously. The role of medicinal plants is particularly important in the Himalayan region [6]. These areas are richly endowed with a variety of plant species, many of which have medicinal properties.

*Eremurus himalaicus *Baker commonly known as Himalayan Desert Candle is a wildornamental herb of Liliaceae family found on rocky slopes of the drier areas of Himalayas that can be easily identified by its tall stout spike-like cluster of hundreds of white flowers with protruding orange anthers [7]. It is locally known as Hulla, Kaihloon, Dharshaag, Chhil haak, and Bulkutor Yalun. Till date no scientific research work has been done on the medicinal values of this plant and the present study is the first attempt to scientifically assess the hypoglycaemic potential of* Eremurus himalaicus.*

## 2. Material and Methods

### 2.1. Plant Material Collection

The plant* Eremurus himalaicus* was collected from FakirGoojree area of Dhara, Srinagar, in the month of May and was identified and authenticated from The Centre for Biodiversity and Taxonomy, Department of Botany, University of Kashmir under voucher specimen number 1765 (KASH). The plant material was cleaned and dried under shade at room temperature and ground in a grinding mill.

### 2.2. Preparation of Plant Extracts

The ground plant material was successively extracted withPetrol, EtOAc, MeOH, and water in a Soxhlet extractor. The recovered extracts were then reduced in a rotary evaporator and finally stored in airtight containers at 4°C for further use.

### 2.3. Preliminary Phytochemical Analysis of the Extracts

The extracts so obtained weresubjected to preliminary phytochemical screening as follows.

#### 2.3.1. Tannins

To 2 mL of aqueous extract, 2 mL of 5% FeCl_3_ was added and observed for theformation of yellow brown precipitate [8].

#### 2.3.2. Alkaloids

To the 2 mL MeOH filtrate, 1.5 mL of 1% HCl was added. After heating thesolution in water bath, 6 drops of Mayor's reagents/Wagner's reagent/Dragendroff reagent were added. Formation of orange precipitate was observed to detect the presence of alkaloids [9].

#### 2.3.3. Saponins

Aqueous extract of 2 g powder was made and the solution was shaken vigorously andobserved for a stable persistent froth. The frothing was mixed with few drops of olive oil and shaken vigorously after which it was observed for the formation of an emulsion [10].

#### 2.3.4. Cardiac Glycosides

To 2 mL alcoholic filtrate, 1 mL glacial acetic acid and 1-2 drops of FeCl_3_ were added followed by 1 mL of concentrated H_2_SO_4_. A brown ring at the interface indicated the presence of a deoxysugar characteristic of cardenolides. A violet ring may appear below the brown ring, while in the acetic acid layer a greenish ring may form just above the brown ring and gradually spread throughout this layer [11].

#### 2.3.5. Terpenes

To 2 mL of aqueous extract, 5 mL CHCl_3_, 2 mL acetic anhydride, and concentrated H_2_SO_4_ were added carefully to form layer. Reddish brown coloration of interface was observed to detect the presence of terpenes [12].

#### 2.3.6. Flavonoids

2 g plant material was extracted in 10 mL alcohol or water. To 2 mL filtrate, few drops of concentrated HCl followed by 0.5 g of zinc or magnesium turnings were added. The solution was observed for the appearance of magenta red or pink colour after 3 min [8].

#### 2.3.7. Phenolics

To 2 mL of alcoholic or aqueous extract, 1 mL of 1% ferric chloride solution wasadded. Blue or green colour indicated phenols [13].

#### 2.3.8. Anthraquinones

0.5 g of the extract was boiled with 10 mL of H_2_SO_4_ and filtered whilehot. The filtrate was shaken with 5 mL of CHCl_3_. The CHCl_3_ layer was pipetted into another test tube and 1 mL of dilute ammonia was added. The resulting solution was observed for colour changes [12].

### 2.4. Physicochemical Parameters

The various physicochemical parameters that were determined as per The Unani Pharmacopeia of India [14] include the following.

#### 2.4.1. Description

It included evaluation of plant by colour, odour, taste, size, shape, andspecial feature, like touch, texture, and so forth.

#### 2.4.2. Loss on Drying

10 g of plant material was placed (without preliminary drying) afteraccurately weighing it in a tarred evaporated dish. This was dried at 105°C for 5 h and weighed. Drying and weighing was continued at 1 h interval until we got the constant weight. Constant weight was reached when two consecutive weights, after drying for 30 min. and cooling for 30 min. in a desiccator, showed not more than 0.1 g difference.

#### 2.4.3. Extractive Values (Successive)

A known amount of plant material was taken and all the sugars were leached out with cold water, dried thoroughly in a desiccator till weight was constant, and then extracted successively with petrol, EtOAc, MeOH, and water in a Soxhlet extractor for complete extraction and different extracts were weighed quantitatively and percentage with respect to the weight of the plant material taken was calculated.

#### 2.4.4. Total Ash Value

About 2-3 g of ground plant material was incinerated in a tarred platinum/silica crucible at a temperature not exceeding 450°C until free from carbon. Then it was cooled and weighed. The percentage of ash with reference to the air dried plant material was calculated.

#### 2.4.5. Acid Insoluble Ash Value

To the crucible containing total ash, 25 mL of dilute HCl was added. The insoluble matter was collected on an ashless filter paper (Whatmann number 41) and washed with hot water until the filtrate was neutral. The filter paper containing insoluble matter was transferred to the original crucible and dried on a hot plate and ignited to constant weight. The residue was allowed to cool in a suitable desiccator for 30 min. and weighed without delay. The content of the insoluble ash was calculated with reference to the air dried plant material.

#### 2.4.6. Water Soluble Ash Value

The ash was boiled for 5 min with 25 mL of water; insoluble matter was collected in a Gooch crucible or on an ash less filter paper, washed with hot water, and ignited for 15 min. at a temperature not exceeding 450°C. The difference in the weight of the insoluble matter and the weight of ash represented the water soluble ash. The percentage of water soluble ash was calculated with reference to the air dried plant material.

#### 2.4.7. Residue on Ignition/Sulfated Ash Test

A platinum/silica crucible was heated to redness for 10 min, allowed to cool in a desiccator, and weighed. Accurately weighed 1-2 g of the plant material was put into the crucible, gently ignited at first, until the substance was thoroughly charred. The residue was cooled, moistened with 1 mL of H_2_SO_4_, heated gently until white fumes were no longer evolving, and ignited at 800°C ± 25°C until all black particles disappeared. The ignition was conducted in a place protected from air currents. The crucible was allowed to cool; a few drops of H_2_SO_4_ were added and the crucible was heated. Then it was ignited as before, allowed to cool, and weighed. The operation was repeated until two successive weighing did not differ by more than 0.5 mg.

#### 2.4.8. pH Value at 10% and 1% Dilution

*(1) pH of 10% Solution*. An accurately weighed 10 g of drug was dissolved in accuratelymeasured 100 mL of water and filtered and the pH of filtrate was checked with a standardized glass electrode. 

*(2) pH of 1% Solution*. An accurately weighed 1 g of drug was dissolved in accuratelymeasured 100 mL of water and filtered and the pH of filtrate was checked with a standardized glass electrode.

### 2.5. Experimental Animals

Healthy adult Wistar strain male albino rats weighing 190–220 g were obtained from Regional Research Institute of Unani Medicine (RRIUM), Srinagar. The animals were kept under standard conditions. Animal studies had approval of IAEC, RRIUM, and Srinagar.

### 2.6. Acute Toxicity Testing

The study was performed as per the Organization for EconomicCooperation and Development (OECD) guidelines number 425.

### 2.7. Effect of Different Extracts of* Eremurus himalaicus* on Normal Rats

It comprised of two tests.

#### 2.7.1. Oral Glucose Tolerance Test

The oral glucose tolerance test was performed in overnight fasted (18 h) normal rats. Healthy rats were randomly selected and distributed into five groups (*n* = 6). One of those groups was administered distilled water and the rest four groups were given orally EtOAc, MeOH, and aqueous extracts of* Eremurus himalaicus* (500 mg/kg body weight, resp.) and glibenclamide (10 mg/kg body weight). Glucose (2 g/kg body weight) was fed 1 h after the administration of extracts and glibenclamide. Blood was withdrawn from the tail vein at 0, 60, 90, 120, and 150 min of glucose administration and glucose levels were estimated using Accucheck Go blood glucose monitoring kit.

#### 2.7.2. Effect of Different Extracts on Normoglycaemic Rats

Healthy Wistar strain albino ratswere selected and randomly divided into different groups with six animals in each group serving as group “A” = normal control, group “B” = EtOAc, 250 mg/kg body weight; group “C” = EtOAc 500 mg/kg body weight; group “D” = MeOH, 250 mg/kg body weight; group “E” = MeOH, 500 mg/kg body weight; “F” = aq., 250 mg/kg body weight, “G” = aqueous, 500 mg/kg body weight, and “H” = glibenclamide (10 mg/kg body weight). An identification mark was given to the rats of each group using picric acid. The blood glucose level of the rats was measured after overnight fasting. Group “A” was given simple drinking water which served as normal control and rest of the groups were given their respective extracts, mentioned above, orally for a period of 14 days. Blood was collected again on the 7th day and 14th day of dosing, through the retro-orbital sinus of the rats. The serum from the blood was separated and labeled with the animal number. The estimation of glucose level was done on an autoanalyser.

### 2.8. Statistical Analysis

All the values were expressed as mean ± standard deviation (S.D.) andanalyzed for ANOVA and post hoc Dunnett's *t*-test. Differences between groups were considered significant at *P* < 0.001 and *P* < 0.05 levels.

## 3. Results and Discussion

The physicochemical properties revealed that the plant was tall and erect, with medium green spike-like foliage and white inflorescence. The successive extract value of ethyl acetate, methanol and water extracts were found to be 3.10%, 26.12%, and 14.6%. Total ash value of plant material indicated that the amount of minerals and earthy material attached to the plant material and its value was calculated to be 9.702% w/w. The amount of the acid insoluble siliceous matter present in the plant was 8.826% w/w. The water soluble extractive value indicated the presence of sugar, acids, and inorganic compounds. The alcohol soluble extractive values indicated the presence of polar constituents and its value was found to be 1.427% w/w. The value for residue on ignition was 0.963% w/w. The pH values of 1% and 10% solutions were 6.18 and 6.02, respectively. The value for loss on drying was found to be 1.419% w/w; less value of moisture content could prevent bacterial, fungal, and yeast growth (Table 1).

Phytochemical investigation of different extracts of* Eremurus himalaicus* revealed the presence of alkaloids, tannins, saponins, terpenoids, flavonoids, phenolics, and cardiac glycosides as secondary metabolites (Table 2). Many of these compounds have been shown to produce potent hypoglycaemic, antihyperglycaemic, and glucose suppressive activities [15, 16]. These effects might be achieved by facilitating insulin release from beta pancreatic cells, inhibiting glucose absorption in gut, stimulating glycogenesis in liver and/or increasing glucose utilization by the body [15–18].

Acute toxicity studies revealed that the EtOAc, MeOH, and aqueous extracts of* Eremurus himalaicus *were safe up to 2000 mg/kg of body weight and approximate LD_50_ is more than 2000 mg/kg.

60 min prior administration of the extracts (500 mg/kg of body weight) followed by the glucose load did not allow the blood glucose level to go higher as compared to the normal ones (Table 3). The effect was comparable to that of the standard antidiabetic drug, glibenclamide. Maximum effect was observed for aqueous and MeOH extracts.

The evaluation of the effect of extracts on blood glucose levels of normoglycaemic rats revealed the results that are in accordance with that of the results obtained for oral glucose tolerance test; that is, the aqueous extract (250 mg/kg and 500 mg/kg; *P* < 0.001) followed by MeOH extract (500 mg/kg; *P* < 0.05) showed significant decrease in the fasting blood glucose levels of the rats; however, the ethyl acetate extract (250 mg/kg and 500 mg/kg) and the MeOH extract (250 mg/kg) did not show significant lowering in the blood glucose levels of the rats (Table 4). The maximum reduction was shown by standard followed by aqueous extract which might be due to the presence of saponin glucosides that are soluble in water and have a glucagon lowering effect, therefore, might enhance glucose utilization [19]. Another reason for the plasma glucose lowering action may be due to the decreased gluconeogenesis, which appears to be related to the antioxidant properties of the plant extract [20]. Interference with the absorption of dietary carbohydrates in the small intestine and facilitation of utilization of glucose by peripheral tissues mediated by an insulin dependent glucose transporter can be another reason for the hypoglycaemic nature of the aqueous extract of* Eremurus himalaicus* plant [21, 22]. The hypoglycemic effect may also be due to the presence of insulin-like substance found in various plants [23].

## 4. Conclusions

The findings of this study indicate the presence of various phytochemicals in the plant extracts, which may be responsible for the pharmacological activity [24, 25]. The aqueous extract is most potent in decreasing the blood glucose levels in normal rats and it might be producing this effect by a mechanism independent from the insulin secretion, for example, by the inhibition of endogenous glucose production [26] or by the inhibition of intestinal glucose absorption [27].

Extracts of* Eremurus himalaicus* appear to be attractive materials for further studies leading to possible drug development for diabetes which is relatively inexpensive and less time consuming and more suited to our economic conditions than allopathic drug development.

## Figures and Tables

**Table 1:** General physicochemical parameters of *Eremurus himalaicus. *

Test parameters	Results
Description	Medium green foliage, white inflorescence, slightly bitter in taste
Loss on drying at 105°C	1.419% w/w
Successive extract value	
Petrol	2.30%
EtOAc	3.10%
MeOH	26.12%
Aqueous	14.60%
Total ash	9.702% w/w
Acid insoluble ash	8.826% w/w
Water soluble ash	1.427% w/w
Residue on ignition/sulphated ash	0.963% w/w
pH of 1.00% w/v soln.	6.18
pH of 10.00% w/v soln.	6.02

**Table 2:** Phytochemical analysis of extracts of *Eremurus himalaicus. *

Constituents	Results
Alkaloids	+
Tannins	+
Glycosides	+
Saponins	+
Anthraquinones	−
Terpenoids	+
Flavonoids	+
Phenolics	+

+ sign indicates secondary metabolite is present; − sign indicates secondary metabolite is not present.

**Table 3:** Effect of different extracts of* Eremurus himalaicus *and glibenclamide on oral glucose tolerance of rats.

Groups	Blood glucose level (mg/dL)				
	0 min	60 min	90 min	120 min	150 min
Control	81.32 ± 4.44	139.37 ± 5.31	128.37 ± 5.15	120.80 ± 4.65	113.6 ± 4.29
Ethyl acetate extract, 500 mg/kg	81.32 ± 4.26	133.46 ± 5.23	120.19 ± 5.44**	113.79 ± 3.79**	105.76 ± 4.31**
Aqueous extract, 500 mg/kg	83.06 ± 4.93	120.93 ± 2.72*	114.38 ± 2.68*	99.68 ± 6.98*	85.14 ± 3.46*
MeOH extract, 500 mg/kg	84.26 ± 5.08	121.44 ± 7.97*	119.01 ± 4.44**	111.59 ± 5.84**	97.18 ± 5.87*
Glibenclamide, 10 mg/kg	81.93 ± 6.28	112.76 ± 4.00*	103.33 ± 5.49*	89.66 ± 6.57*	75.69 ± 4.38*

Data represented as mean ± S.D values of 6 animals each. *P < 0.001 and **P < 0.05 (Dunnett *t*-test); diabetic control was compared with the normal; extract and standard treated groups were compared with the diabetic control.

**Table 4:** Effect of different extracts of *Eremurus himalaicus* on fasting blood glucose levels of normoglycaemic rats.

Groups	Blood glucose level (mg/dL)			
	0th day	7th day	14th day	% Variation
Normal control	84.01 ± 4.64	82.21 ± 5.58	80.29 ± 6.53	4.43
Ethyl acetate extract, 500 mg/kg	85.00 ± 4.23	82.10 ± 4.60	79.73 ± 4.99	6.20
Ethyl acetate extract, 250 mg/kg	85.14 ± 4.05	84.10 ± 5.14	81.57 ± 6.69	4.22
Aq. extract, 500 mg/kg	81.64 ± 3.72	72.5 ± 3.44	65.58 ± 2.86*	19.67
Aq. extract, 250 mg/kg	81.40 ± 4.08	73.36 ± 2.67	68.51 ± 2.92*	15.83
MeOH extract, 500 mg/kg	81.53 ± 2.38	77.26 ± 1.65	72.74 ± 2.15**	10.78
MeOH extract, 250 mg/kg	84.35 ± 6.21	79.30 ± 4.03	76.08 ± 2.37	9.80
Glibenclamide, 10 mg/kg	84.22 ± 2.26	71.59 ± 2.41*	65.36 ± 3.80*	22.39

Data represented as mean ± S.D values of 6 animals each. *P < 0.001 and **P < 0.05 (Dunnett *t*-test); diabetic control was compared with the normal; extract and standard treated groups were compared with the diabetic control.
